# Rapid improvement of angiostenosis due to isolated middle cerebral artery dissection: A case report

**DOI:** 10.1097/MD.0000000000009695

**Published:** 2018-01-26

**Authors:** Li Zhang, Guangxian Nan, Ying Mao, Lumei Chi

**Affiliations:** Department of Neurology, China-Japan Union Hospital of Jilin University, Changchun, China.

**Keywords:** angiostenosis, anti-platelet therapy, case report, dissection, middle cerebral artery

## Abstract

**Rationale:**

Intracranial arterial dissection is a rare cause of ischemic stroke, and isolated middle cerebral artery dissection (MCAD) is extremely rare, having been described only in sparse case reports. The etiology, clinicoradiological features, and treatment strategies are not yet well understood.

**Patient concerns:**

A 49-year-old man presented with rapidly progressive aphasia and motor disturbance of the right limbs.

**Diagnoses:**

Neuroimaging evaluation confirmed a diagnosis of MCAD and cerebral infarction.

**Interventions:**

The patient underwent oral anti-platelet therapy (100 mg aspirin daily).

**Outcomes:**

The patient recovered to normal status within 2 weeks following antiplatelet treatment. During a follow-up period of 2 years, he remained neurologically asymptomatic and led a virtually normal life.

**Lessons:**

It is crucial for clinicians to be aware of this entity, as the diagnosis of MCAD is quite challenging. Antiplatelet therapy is effective for treating this condition, and the prognosis can be favorable.

## 1 Introduction

Intracranial artery stenosis or occlusion is a frequent disease, which is implicated in 33% to 50% of stroke cases, and intracranial atherosclerosis is the most common diagnosis in these cases.^[1]^ Intracranial arterial dissection is a rare cause of intracranial artery stenosis or occlusion; especially, isolated middle cerebral artery dissection (MCAD) is extremely rare. MCAD was first identified in 1915 and since then has only been described in sparse case reports.^[2]^ The early diagnosis of MCAD is challenging, and this disease is associated with high disability and mortality. Herein, we report a case of MCAD and cerebral infarction in which effective antiplatelet therapy led to rapid recovery.

## 2 Case report

**Figure 1:**
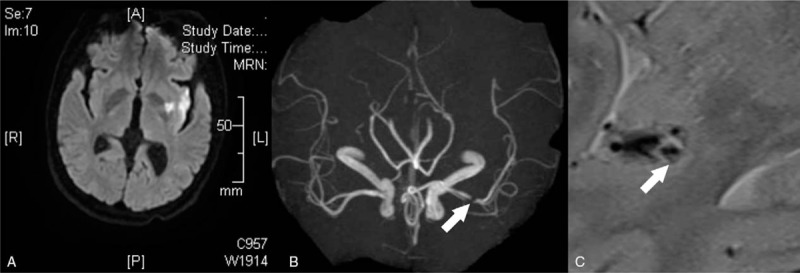
Brain diffusion-weighted magnetic resonance imaging revealed focal hyperintensities in the left insula and basal ganglia (A). Head magnetic resonance angiography showed an angiostenosis (arrow) in the initial part of the middle cerebral artery (B). MRI of the arterial walls revealed a double-lumen sign (C, arrow).

**Figure 2:**
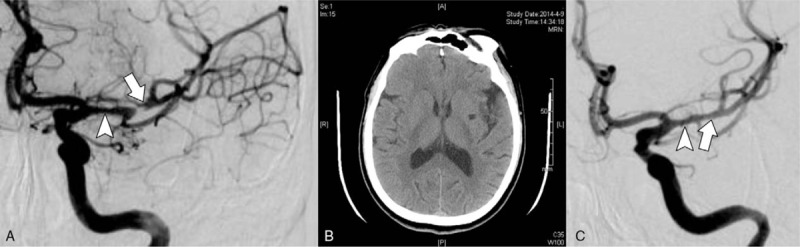
Brain digital subtraction angiography at admission revealed a remarkable stenosis (arrow) and a slight stenosis (arrowhead) in the left middle cerebral artery (A). Eleven days following anti-platelet treatment, the repeated brain CT showed hypodensity in the left putamen, suggestive of an old cerebral infarction (B). Repeated DSA (C) showed that the angiostenosis in the middle cerebral artery was significantly improved (arrow), and only slight stenosis remained (arrowhead).

A 49-year-old man presented with a 3.5-hourcourse ofprogressive aphasia and motor disturbance of the right limbs. He also complained of numbness in the right facial region. Brain computed tomography (CT) was requested in the local institution, and eventually a diagnosis of cerebral infarction was made. The patient was subsequently transferred to our department. Review of systems was negative for unconsciousness, headache, nausea, vomiting, coughing, or dysphagia. Laboratory data were within normal limits. No sphincter dysfunction was noted. The patient reported a known history of hypertension for 2 months, but he had not taken antihypertensive medicine regularly. The blood pressure at admission was normal (135/85 mm Hg). Physical examination showed incomplete motor aphasia, right facial nerve paralysis, weakness of the right limbs, right hemidysesthesia, and a positive pathologic reflex. The proximal and distal muscle strengths of the right upper limb were grade 0 and 2, respectively, and the muscle strength of the right lower limb was grade 4. The patient's neurological function was graded as 6 points according to the National Institutes of Health Stroke Scale (NIHSS) and 4 points according to the modified Rankin Scale (mRS). The brain CT scan (February 15, 2014) showed poorlydefined, patchy hypodense lesions in the left basal ganglia and corona radiata. Brain diffusion-weighted magnetic resonance imaging (MRI-DWI) revealed spot-like and patchy hyperintensities in the left insula, basal ganglia, putamen, corona radiata, and centrum semiovale (Fig. 1A). Further head magnetic resonance angiography (MRA) was requested on February 16, 2014, which showed local stenosis. The remarkable stenosis covering more than 95% of the lumen area was located in the initial part of middle cerebral artery (Fig. 1B). MRI (February 16, 2014) of the arterial walls revealed a double-lumen sign (Fig. 1C). In addition, brain digital subtraction angiography (DSA; February 17, 2014) confirmed the angiostenosis in the initial part of middle cerebral artery (Fig. 2A). A diagnosis of cerebral infarction was made, and the cause was considered to be left MCAD. An oral antiplatelet regimen was scheduled (100 mg aspirin daily). Eleven days later, the patient's symptoms were completely relieved without any notable neurological sequelae. The NIHSS and mRS scores were both improved to 0 points. Repeated brain CT showed hypodensity in the left putamen, suggestive of an old cerebral infarction (Fig. 2B). Repeated DSA showed that the angiostenosis in the middle cerebral artery was significantly improved (Fig. 2C). During a follow-up period of 2 years, the patient remained neurologically asymptomatic and led a virtually normal life.

## 3 Discussion

Isolated MCAD has been rarely reported as a cause for stroke, and most of the reported cases were in young adults.^[2]^ Since the first case of isolated MCAD was described by Turbull et al^[2]^ in 1915, there has been no randomized controlled trial, and this disease remains poorly understood. The definite incidence of isolated MCAD is still unclear; according to the literature, a combination of low clinical suspicion and inadequate understanding of the clinicoradiological characteristics may contribute to the low incidence of isolated MCAD.

The causes of cerebral artery dissection are still undetermined and may be multifactorial. The identified predisposing risk factors include history of trauma and connective tissue related disorders such as fibromuscular dysplasia, cystic medial necrosis, moyamoya disease, Marfan syndrome, and Ehlers–Danlos syndrome type IV. Common vascular risk factors, including hypertension, smoking, diabetes mellitus, hyperlipidaemia, and oral contraceptives, have also been implicated in the pathogenesis of cerebral artery dissection. However, the majority of patients with artery dissection may be cryptogenic.

Intracranial arterial dissection is more frequently found in the posterior circulation and is less common in the anterior circulation, and that occurring solely in the middle cerebral artery is extremely rare. Regarding the dynamic pathological process of MCAD, it is hypothesized that the hemodynamic disturbances might result from stenosis or occlusion from a lumen wall hematoma with subsequent thrombus formation and distal embolism, and this dysfunction could lead to an ischemic stroke eventually.^[3]^

The clinical manifestations of MCAD are variable. The focal neurological deficiencies are usually nonspecific, and the most common is headache.^[2,4]^ According to a previously published systematic review, more than 40% of all MCAD patients can present with headache, and nearly 20% can present without any observed neurological deficits. The patient in the present case did not experience headache, and the neurological symptoms were consistent with the infarction location. In addition, when the vascular adventitia is damaged in MCAD, the blood can flow out the lumen, causing intracerebral hemorrhage or subarachnoid hemorrhage.

Recognition of MCAD mainly depends on comprehensive radiological profiles. As reported in the literature, the most commonly used imaging modality for diagnosis of MCAD is DSA (75.4%), followed by CT or CTA (72.1%), MRI or MRA (62.3%), pathological evidence (9.8%), and transcranial Doppler (1.6%).^[5]^ DSA is the gold standard for diagnosis; however, the radiation and risk of perioperative complications have led to widespread application of other imaging techniques. Although high-resolution MRI is an advanced alternative noninvasive method, it still has several technical limitations such as overestimation of stenosis and artifacts. CT also has limitations due to its ionizing radiation and disability in usage as a vessel-monitoring system.^[1]^ In the present case, DSA revealed local stenosis, suggesting a possible diagnosis of artery dissection. The double-lumen sign on MRI of arterial walls also provided suggestive clues. However, some MCADs remain undetectable on neuroimaging, and the eventual diagnosis still requires histopathological evidence.

Due to the rarity of this condition, the optimal therapeutic option is still unclear. Currently, the treatment for intracranial artery dissection is based on the experience in extracranial artery dissection management, including conservative observation, antiplatelet, anticoagulation, thrombolysis, surgery, and endovascular interventions.^[4]^ To date, no study has been performed that compares the efficacy of different alternatives. The safety of thrombolysis for intracranial artery dissection has not been verified. Although thrombolysis can reverse the ischemic pathological process of MCAD, it may also lead to hematoma enlargement, formation of pseudoaneurysm, and subarachnoid hemorrhage, and thus, the application of thrombolysis should be prudent. The role of anticoagulation and antiplatelet therapy is still under debate. Anticoagulation also has the risk of hematoma enlargement and subarachnoid hemorrhage, and thus, should not be considered as the first-line choice. Intracranial stent deployment can be proposed for patients who do not respond after medical treatment in the event of spontaneous MCAD. In our case, a diagnosis of MCAD and focal cerebral infarction was made, and we planned a solely antiplatelet regimen. After antiplatelet treatment, the patient experienced recovery rapidly, and he got a favorable prognosis at the 2-year follow-up. The clinical and radiological improvement in our patient supports our decision to administer antiplatelet treatment. In the literature, the rapid improvement of angiostenosis due to isolated MCAD has not been reported. Notably, recurrence of MCAD occurs frequently within several weeks following onset. Although our patient was event-free 2 years later, a much longer follow-up is still necessary.

## 4 Conclusion

Angiostenosis and cerebral infarction caused by isolated MCAD is extremely rare. It is crucial for clinicians to be aware of this entity as the diagnosis of MCAD is quite challenging. Antiplatelet therapy can be effective in some patients, and the prognosis can be favorable.
